# Luminescence Enhancement of an Indium(III) Complex Through Introducing an Electron-Donating Group in (1*H*-Pyrazol-1-yl)pyridazine

**DOI:** 10.3390/molecules31183217

**Published:** 2026-09-11

**Authors:** Evgeniia Sergeevna Sedykh, Nikita Vasilievich Naletov, Veronika Igorevna Komlyagina, Yulia Sergeevna Spiridonova, Elvira Ilgizovna Musina, Marianna Ivanovna Rakhmanova, Nikolay Filippovich Romashev, Katerina Aleksandrovna Vinogradova, Marat Damirovich Nafikov, Alexey Yuryevich Vorob’ev, Iakov Sergeevich Fomenko, Artem Leonidovich Gushchin

**Affiliations:** 1 Department of Natural Sciences, Novosibirsk State University, 1 Pirogova Str., Novosibirsk 630090, Russian.naletov@g.nsu.ru (N.V.N.);; 2 Nikolaev Institute of Inorganic Chemistry, Siberian Branch of Russian Academy of Sciences, 3 Acad. Lavrentiev Ave., Novosibirsk 630090, Russia; 3 Arbuzov Institute of Organic and Physical Chemistry RAS, Kazan 420088, Russia; 4 N. N. Vorozhtsov Novosibirsk Institute of Organic Chemistry, Siberian Branch of Russian Academy of Sciences, 9 Acad. Lavrentiev Ave, Novosibirsk 630090, Russia; vor@nioch.nsc.ru

**Keywords:** indium, pyrazolyl–pyridazine, morpholine, photoluminescence, excitation-dependent emission, crystal structure, TD-DFT calculations

## Abstract

New deep-blue-emitting materials are crucial for Organic Light-Emitting Diode (OLED) technology, as iridium-based blue emitters often suffer from degradation and inadequate colour purity. Indium(III) complexes offer an alternative, since the d^10^ configuration of In^3+^ precludes metal-centred transitions, and emission arises from ligand-centred states via the chelation-enhanced fluorescence (CHEF) effect. We previously reported complexes **1** ([In(L^H^)(H_2_O)Cl_3_]) and **2** ([In(L^Me^)_2_Cl_2_][InCl_4_]), which exhibited excitation-dependent emission, and the electronic transitions were assigned as ILCT for **1** and mixed ILCT/LL’CT for **2**. Herein, we present a new complex, complex **3**, i.e., [In(L^Morph^)_2_Cl_2_][InCl_4_], where L^Morph^ contains electron-donating morpholine substituents, in contrast to the acceptor chloride group in L^H^ and L^Me^. Complex **3** was characterised by elemental CHN analysis, infrared spectroscopy (IR), nuclear magnetic resonance spectroscopy (NMR), ultraviolet–visible spectroscopy (UV-Vis), single-crystal X-ray diffraction analysis (SC-XRD), and photoluminescence. The ionic structure of **3** is analogous to **2**, with a *cis*-octahedral [In(L^Morph^)_2_Cl_2_]^+^ cation and tetrahedral [InCl_4_]^−^ anion. The UV-Vis spectrum of **3** shows a significant bathochromic shift relative to **1** and **2** which is attributable to the morpholine group. TD-DFT calculations were employed to assign the electronic transitions. The introduction of a morpholino group into the pyridazine ring resulted in significant changes to the solid-state photoluminescence properties of the indium complex: (i) the emission maximum shifted to the red region (CIE 1931 coordinates: 0.1725, 0.2487), (ii) the lifetime increased by an order of magnitude, and (iii) the quantum yield rose to 15%. This work highlights that substituent variation on the pyrazolyl–pyridazine scaffold provides a versatile route to tune the structural and photophysical properties of indium(III) complexes.

## 1. Introduction

Luminescent indium(III) complexes bearing N,O-donor ligands have emerged as a well-established and extensively investigated class of coordination compounds [1]. Their optical properties can be finely modulated with remarkable precision through systematic variations in the coordination geometry and the ligand environment, offering a versatile platform for the rational design of emissive materials [2,3,4,5,6,7,8,9,10,11,12,13,14]. Furthermore, indium complexes are capable of achieving exceptionally high photoluminescence quantum yields, approaching unity in certain cases, owing to efficient triplet-state emission [15]. This phenomenon is primarily attributed to the heavy-atom effect of indium, which promotes spin–orbit coupling and facilitates efficient intersystem crossing, thereby enabling phosphorescence from ligand-centred excited states. These unique features position indium(III) complexes as promising alternatives to conventional transition-metal-based emitters for applications in optoelectronics and photonics.

Beyond oxyquinolinates, indium(III) complexes with nitrogen-donor chelates [13,16,17,18,19,20,21,22,23], including phthalocyanines [24,25], as well as mixed-ligand systems with N- and O-donor ligands [26,27,28,29,30], have been explored. Dipyrrinate indium(III) complexes show green fluorescence at ~500 nm (quantum yield (QY) ~7%) [22], while substituent engineering can shift emission to 600–650 nm with quantum yields of 30–40% [16]. Salen–indium complexes achieve bright blue luminescence with quantum yields of up to 31.3% [26,31]. Diketiminate indium(III) complexes exhibit fluorescence (550–560 nm, QY up to 30%), sometimes outperforming their aluminium and gallium analogues [13], and, upon fine ligand tuning, they even demonstrate phosphorescence [32]. Tridentate O,N,N-donor Schiff base indium(III) complexes with phenolate-iminopyridine moieties exhibit fluorescence in solution and the solid state (600–700 nm). Electron-withdrawing substituents cause hypsochromic shifts, while electron-donating groups induce bathochromic shifts. These complexes demonstrate moderate quantum yields of up to 8.6% (2.2 ns) in solution and 7.8% (2.9 ns) in the solid state [33]. Related Schiff base systems with similar emission properties have also been reported [34]. In contrast, bis-amidinate indium(III) monochloride complexes bearing aryl-substituted ligands exhibit significantly higher solution-state fluorescence, with quantum yields reaching 45.5% and lifetimes of up to 11 ns [35], demonstrating the potential of amidinate scaffolds for developing more efficient indium-based luminophores. These findings underscore the versatility of indium(III) coordination chemistry and its potential for developing high-performance emitters beyond the traditional Alq_3_ paradigm.

In the present study, we extend our investigation to a new nitrogen-donor ligand, L^Morph^ (4-(6-(3,5-dimethyl-1*H*-pyrazol-1-yl)pyridazin-3-yl)morpholine), for the synthesis of potential luminescent indium(III) complexes. This ligand is selected for several reasons: (i) it can adopt a chelate coordination mode similar to that of previously studied analogues L^H^ and L^Me^ [36]; (ii) it is readily synthesised from commercially available starting materials; and (iii) the morpholine moiety, as a strong electron donor, is expected to significantly perturb the electronic structure of the ligand, potentially leading to enhanced emission properties. Notably, while (1*H*-pyrazol-1-yl)pyridazine ligands have been successfully employed in the synthesis of transition metal complexes, such as manganese(II) complexes based on L^Me^, which exhibit green emission in the solid state [37], and rhenium(I) carbonyl complexes with L^H^ showing red emission in dichloromethane solution [38], their application in indium(III) coordination chemistry remains underexplored. In our previous work, we demonstrated that L^H^ and L^Me^ afford indium(III) complexes (**1** and **2**, respectively) with distinctly different coordination motifs—neutral mononuclear vs. ionic bis-chelate—and excitation-dependent emission properties [36]. The introduction of the morpholine group in L^Morph^ represents a further step towards understanding the structure–property relationships in this class of compounds, as the electron-donating character of the morpholine substituent is expected to induce significant changes in the ligand-centred electronic transitions and, consequently, in the luminescence behaviour of the resulting complex.

The influence of electronic effects on photoluminescence (PL) properties has been intensively studied, and a huge number of works dedicated to donor–acceptor systems («D-A structures» or «D-A-D structures») have been published in recent decades [39,40,41]. The combination of one molecule electron donor and electron acceptor moieties has led to the design of luminescent materials with high quantum efficiency and/or to thermally activated delayed fluorescence (TADF) materials [42,43,44]. Less common in the literature are studies that consider only the effects of donor groups [45,46,47]. It is known that donor groups decrease the HOMO-LUMO gap, which shifts the emission spectrum to the red region. In [48], it is shown that donor substituents can lead to double emission, and, in [49], they lead to mechanochromic behaviour. Most of these studies have been carried out on organic molecules. Investigations in which the influence of donor substituents on the PL properties of coordination compounds is studied are significantly fewer in number; such reports are mainly available for the most intensively studied classes of luminescent complexes—iridium(III) [50], copper(I) [51], or ruthenium(II) [52] complexes—or, in the case of one work, a dedicated derivative of Alq_3_ [53].

In the current study, we report the synthesis and comprehensive characterisation of a new indium(III) complex, [In(L^Morph^)_2_Cl_2_][InCl_4_] (complex **3**), obtained from the reaction of InCl_3_ with L^Morph^. The complex was fully characterised by CHN, IR, NMR, and UV-Vis absorption spectroscopies, as well as single-crystal X-ray diffraction and photoluminescence spectroscopy. The luminescence properties of **3** were investigated both in the solid state and in an MeCN solution. A detailed comparative analysis with previously reported complexes **1** and **2** is presented, highlighting the influence of the morpholine substituent on the structural features, electronic transitions, and photophysical properties.

## 2. Results and Discussion

**Figure 1:**
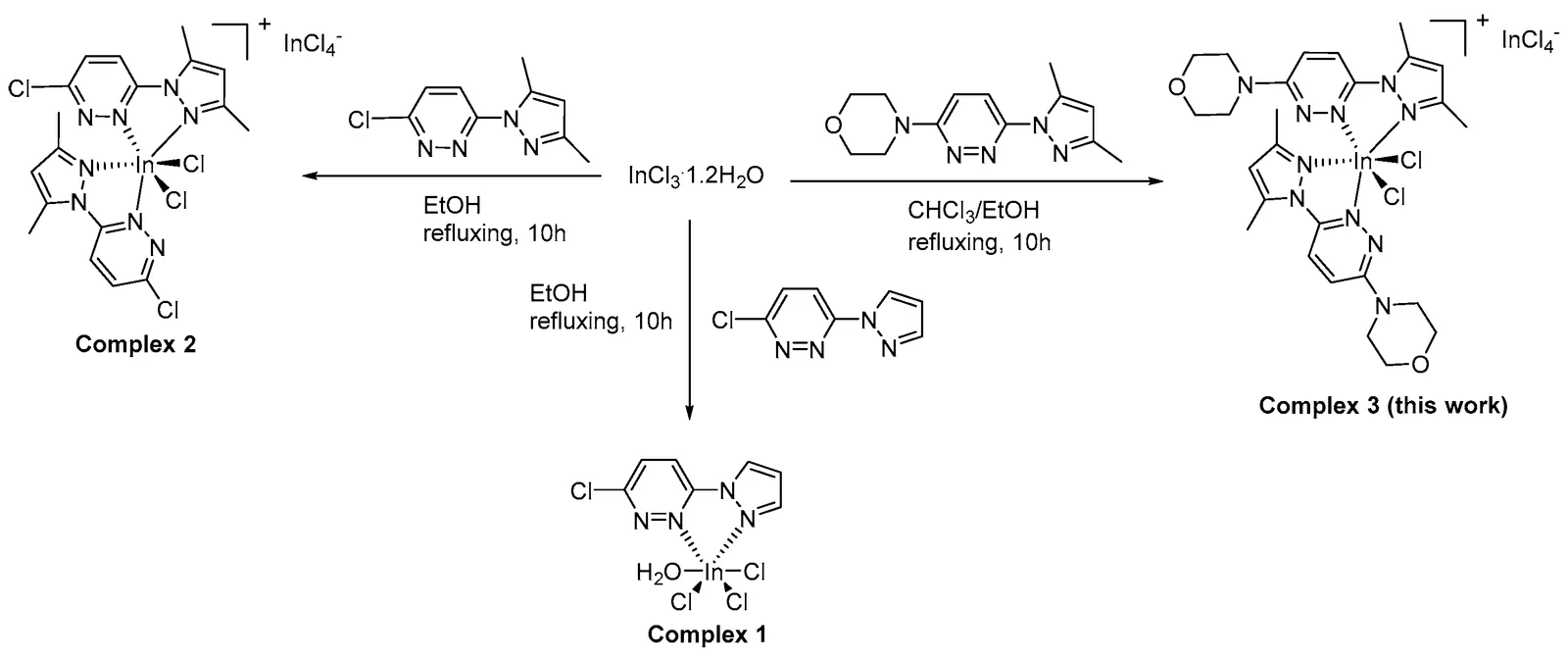
Synthesis of complexes [In(L^H^)(H_2_O)Cl_3_] (**1**), [In(L^Me^)_2_Cl_2_][InCl_4_] (**2**) [36], and [In(L^Morph^)_2_Cl_2_][InCl_4_] (**3**) (this work).

The interaction of indium trichloride with L^Morph^ leads to the formation of complex [In(L^Morph^)_2_Cl_2_][InCl_4_] (**3**). Complex **3** is an analogue of previously obtained compounds [In(L^H^)(H_2_O)Cl_3_] (**1**) and [In(L^Me^)_2_Cl_2_][InCl_4_] (**2**), which are described in [36]. Although complexes **1**–**3** are obtained via analogous synthetic routes (Figure 1), they differ fundamentally in the nature of the substituents appended to the pyrazolyl–pyridazine ligand backbone. Complexes **1** and **2** are distinguished by the presence of methyl groups in the pyrazole ring, whereas complex **3** contains a morpholine fragment instead of the electron-withdrawing chloride substituent present in the parent ligand scaffold (L^H^ and L^Me^). The incorporation of this electron-donating morpholine moiety induces substantial modifications in the electronic structure of the ligand, which, in turn, leads to marked differences in the optical properties of complex **3** relative to those of complexes **1** and **2**.

**Figure 2:**
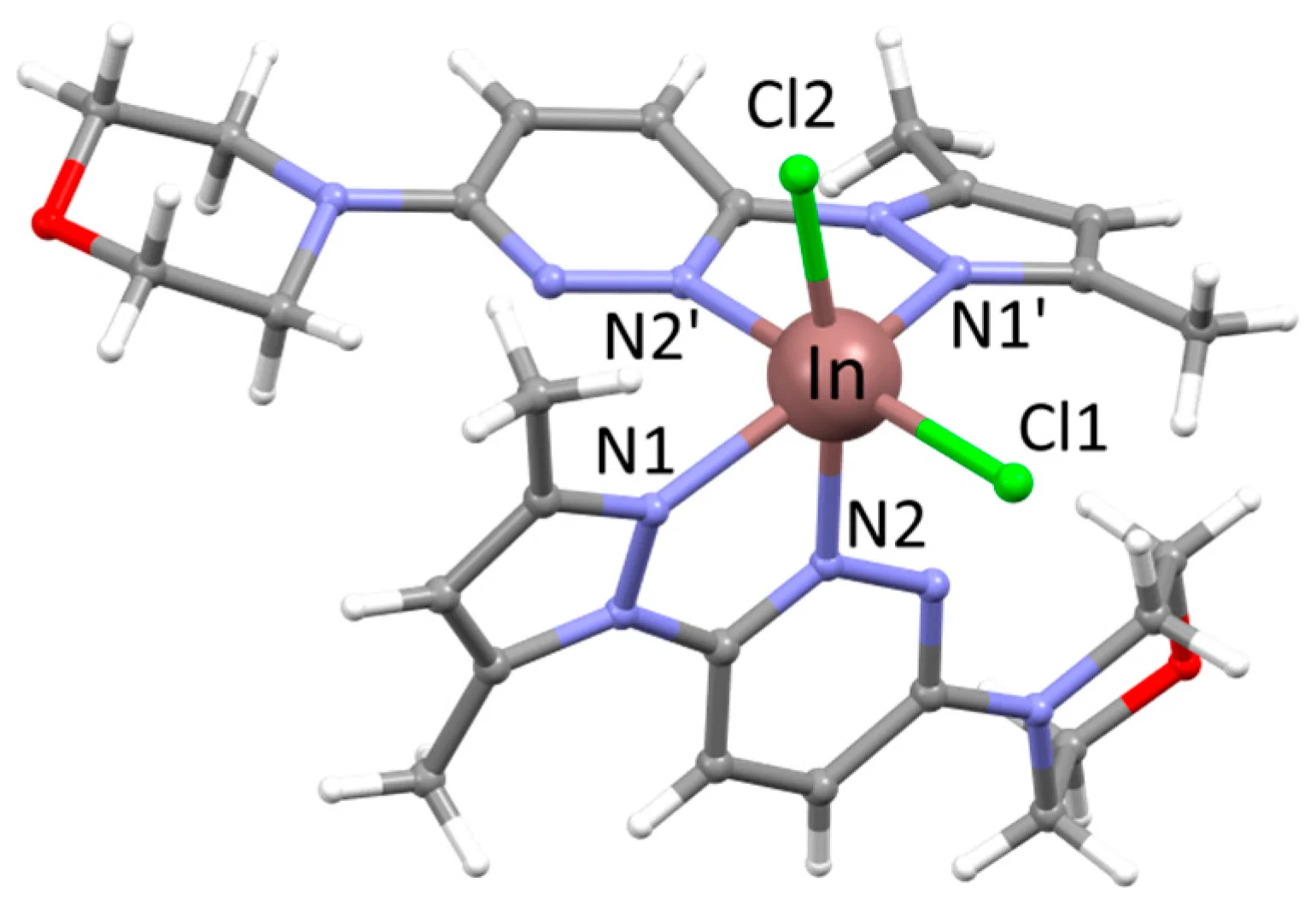
The molecular structure of **3**.

Single crystals of **3**·CHCl_3_ suitable for X-ray diffraction analysis were obtained, and the crystal structure of **3**·CHCl_3_ was determined. The molecular structure of **3** is shown in Figure 2, and selected bond lengths are summarised in Table 1. Compound **3**·CHCl_3_ has an ionic structure consisting of a cationic part [In(L)_2_Cl_2_]^+^ and a tetrahedral InCl_4_^−^ anion, along with one chloroform solvent molecule. The structure of the cationic unit of **3** can be described as follows: the central In atom is octahedrally coordinated by two chlorine atoms and four nitrogen atoms from two pyrazolyl–pyridazine ligands. The geometry of the chloride ligands (Cl–In–Cl = 98.26(4)°) indicates the presence of the *cis*-isomer in the cation of complex **3**. The bond lengths In–N1 (2.278(3) Å), In–N2 (2.294(3) Å), In–N1′ (2.255(3) Å), and In–N2′ (2.310(3) Å) are comparable to those reported for our previously described analogous complex [36]. In addition, the dihedral angles between the pyrazole and pyridazine rings are 10.60° and 19.56° for the N1N2 and N1′N2′ ligand fragments, respectively, which may be due to crystal packing effects. In the crystal structure of **3**·CHCl_3_, intermolecular H-π stacking interactions are found between the pyridazine rings (3.41 Å), resulting in the formation of a zigzag motif along the [001] direction (Figure S1).

The molecular geometry of complex **3** is very similar to that of previously reported analogous complex **2** [36] (Figure 1). A comparison of the crystal structures of complexes **2** and **3** revealed that both compounds are isostructural and feature the same ionic motif, [In(L)_2_Cl_2_][InCl_4_], with a *cis*-octahedral cation. The In–N bond lengths in **3** (2.255–2.310 Å) and **2** (2.275–2.314 Å) are nearly identical, as are the In–Cl distances, which show no significant asymmetry in either complex. The Cl–In–Cl angle of 98.26(4)° in **3** confirms the *cis*-geometry, which is consistent with that observed for **2**. However, the dihedral angles between the pyrazole and pyridazine rings differ slightly (10.60° and 19.56° for **3** vs. 17.57° for one fragment in **2**), suggesting conformational flexibility due to crystal packing. Additionally, the crystal packing of **3** is distinguished by intermolecular H-π interactions between pyridazine rings (3.41 Å), forming a zigzag chain along the [001] direction, whereas such interactions were not highlighted for complex **2**.

A comparison of complexes **1**–**3** showed that **1** is a neutral molecular complex with *fac*-[In(L^H^)(H_2_O)Cl_3_] coordination, while **2** and **3** are ionic compounds of the type [In(L)_2_Cl_2_][InCl_4_] with a *cis*-octahedral cation. The molecular geometry of complex **3** is very similar to that of the previously reported analogous complex **2** [36] (Figure 1). The In–N bonds in **1** are significantly asymmetric (2.258(2) and 2.350(2) Å), whereas In–N distances (2.255–2.314 Å) in **2** and **3** are more uniform. The In–Cl, bonds in the cationic moieties of **2** and **3** are shorter (ca. 2.38–2.40 Å) and more symmetric than those in **1** (2.4056–2.4604 Å), which also features a coordinated water molecule. The pyrazole–pyridazine rings are coplanar in **1** (3.15°), but have larger dihedral angles in **2** and **3** (10.60–19.56°), reflecting greater conformational flexibility in the bis-ligated ionic species. The crystal packing of **1** is dominated by O–H···Cl hydrogen bonds, while **3** features H-π stacking interactions between pyridazine rings (3.41 Å), which form a zigzag motif, highlighting the role of the ionic nature and lattice solvent effects in directing supramolecular assembly.

**Figure 3:**
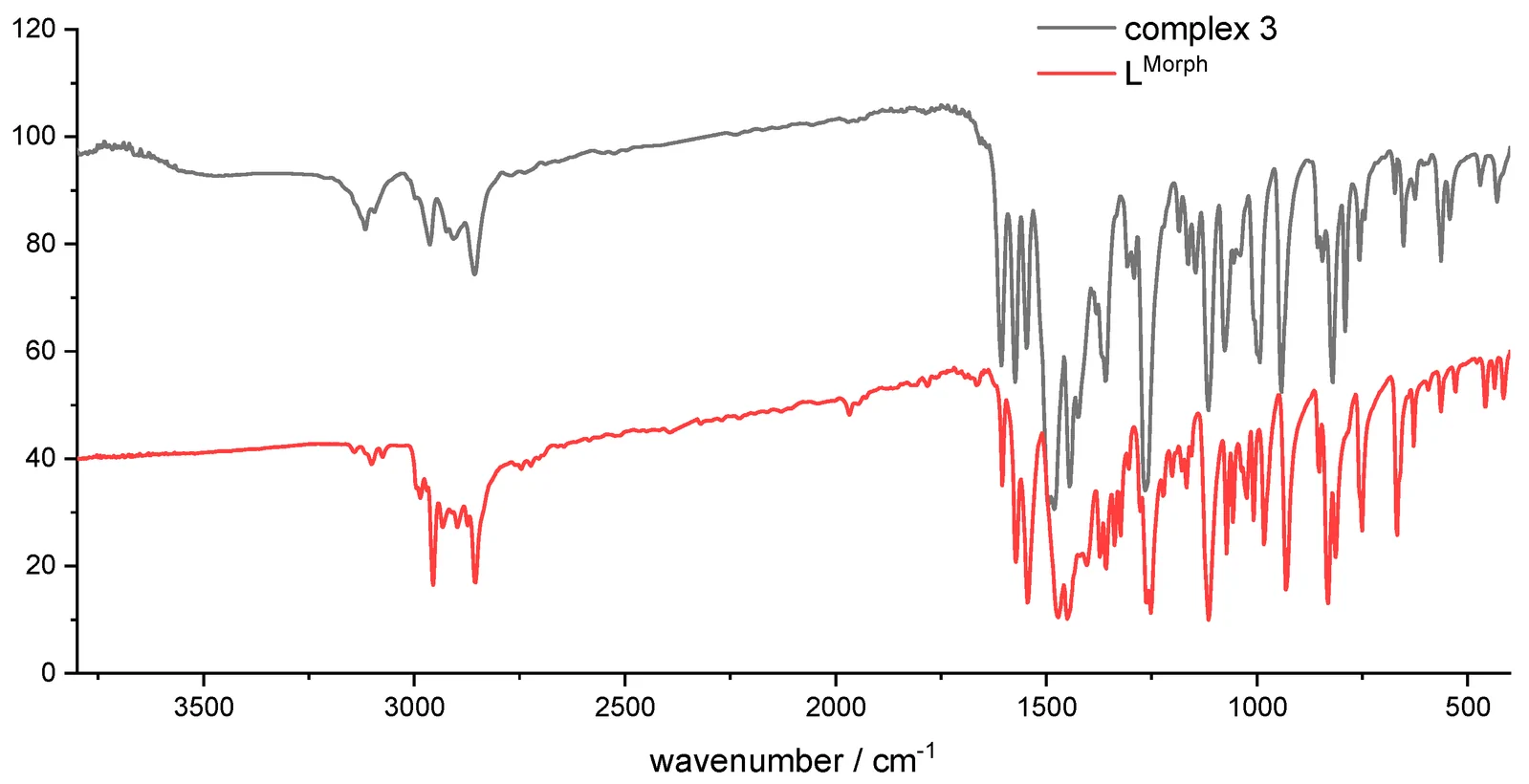
IR spectra of complex [In(L^Morph^)_2_Cl_2_][InCl_4_] (**3**) and L^Morph^.

The IR spectrum of complex [In(L^Morph^)_2_Cl_2_][InCl_4_] (**3**) shows characteristic C–H stretching bands in the 2856–3118 cm^−1^ region, with aliphatic ν(C–H) bands at 2856–2965 cm^−1^ and aromatic ν(=C–H) at 3118 cm^−1^. The bands associated with the C=N and C=C stretching vibrations of the pyrazolyl–pyridazine skeleton are observed within the 1424–1610 cm^−1^ range. The morpholine fragment is confirmed by strong ν(C–O–C) bands at 1262 and 1116 cm^−1^. The methyl substituents are clearly identified by their bending modes at 1445 (δ_as_) and 1361 cm^−1^ (δ_s_). The out-of-plane γ(C–H) deformations of the aromatic rings appear in the 757–996 cm^−1^ region. The coordination of the ligand through nitrogen atoms is supported by weak ν(In–N) bands at 651 and 674 cm^−1^. The characteristic ν(In–Cl) stretching vibrations of the outer-sphere [InCl_4_]^−^ anion are observed at 538 and 564 cm^−1^, while the inner-sphere In–Cl vibrations of the cationic moiety appear at 428 and 468 cm^−1^ (Figure 3).

The ^1^H NMR spectra of the free ligand L^Morph^ and its indium(III) complex **3** were recorded in CD_3_CN at room temperature, and the data are summarised in Table S2. The spectrum of the free ligand L^Morph^ (Figure S2) exhibits the following characteristic signals: two doublets at 7.74 and 7.21 ppm, assigned to the aromatic protons of the pyridazine ring; a broad singlet at 6.03 ppm corresponding to the pyrazole H4 proton; two multiplets at 3.78 and 3.56 ppm, attributed to the morpholine –N–CH_2_– and –O–CH_2_– protons, respectively; and two singlets at 2.53 and 2.21 ppm, corresponding to the two inequivalent methyl groups on the pyrazole ring. The spectrum of complex **3** shows a distinct downfield shift of all aromatic and heterocyclic protons, consistent with the electron-withdrawing effect of the metal centre and the reduced electron density on the ligand upon complexation (Figure S3). Specifically, the pyridazine protons appear at 7.93 and 7.39 ppm, while the pyrazole H4 proton is observed as a broad singlet at 6.49 ppm. The morpholine protons remain as two multiplets at 3.65 and 3.32 ppm, with a slight upfield shift relative to the free ligand. Notably, the methyl protons in the free ligand appear as two separate singlets (2.52 and 2.20 ppm), reflecting their chemical inequivalence, whereas, in complex **3**, they are down-shifted to 2.66 and 2.63 ppm.

The ^1^H NMR spectrum of complex **3** is generally similar to that of the previously reported complex **2**, with both showing downfield shifts of the aromatic and heterocyclic proton signals upon coordination [36]. However, a notable difference is observed for the methyl groups: they appear as two separate singlets at 2.68 and 2.63 ppm for complex **2**, whereas a single signal at 2.63 ppm is observed for **3**.

The experimental X-ray diffraction pattern of complex **3** (Figure S4) differs significantly from the theoretical diffraction pattern calculated from single-crystal diffractometry data. This discrepancy is primarily attributed to the presence of a chloroform solvate molecule in the crystalline lattice, which is readily lost upon exposure to ambient conditions, leading to lattice collapse or a phase transformation that alters the long-range periodicity and, consequently, the observed diffraction profile.

### Photophysics and DFT Calculations

The absorption spectrum of the free ligand L^Morph^ in MeCN solution reveals an intense band at 274 nm and a broad weak shoulder at 317–370 nm. When L^Morph^ is coordinated to indium(III), a distinct bathochromic shift is observed in the spectrum of complex **3**: the main band appears at 286 nm, and the shoulder is in the range of 348–400 nm (Figure S5). This red shift of approximately 12 nm for the main band and 30 nm for the low-energy shoulder indicates a decrease in the HOMO–LUMO gap upon ligand coordination, consistent with the involvement of the nitrogen-donor atoms in metal–ligand bond formation. Comparison with complexes **1** and **2**, for which the main absorption bands are found at 260 nm with shoulders at 282–325 nm and 289–337 nm, respectively, showed that the absorption for complex **3** is significantly shifted to the red region of the spectrum (by ca. 26 nm relative to the main band of **1** and **2**) [36]. This observation is attributed to the presence of the electron-donating morpholine moiety in the ligand of complex **3**, which increases the electron density of the π-system and stabilises the excited state to a greater extent than the chloride substituent in **1** and **2**. Thus, the absorption data highlight the profound influence of the electronic structure of the substituents on the photophysical properties of this class of indium(III) complexes.

The gas-phase geometry of the cation of complex **3** and the free ligand was optimised using the XRD structure as a starting point (Table 2). The absence of imaginary vibrational frequencies confirmed that the obtained stationary points correspond to true minima on the potential energy surface. Subsequent optimisation in acetonitrile did not lead to a significant change in the structure of **3** and ligand, indicating that the coordination geometry is largely retained in solution.

**Figure 4:**
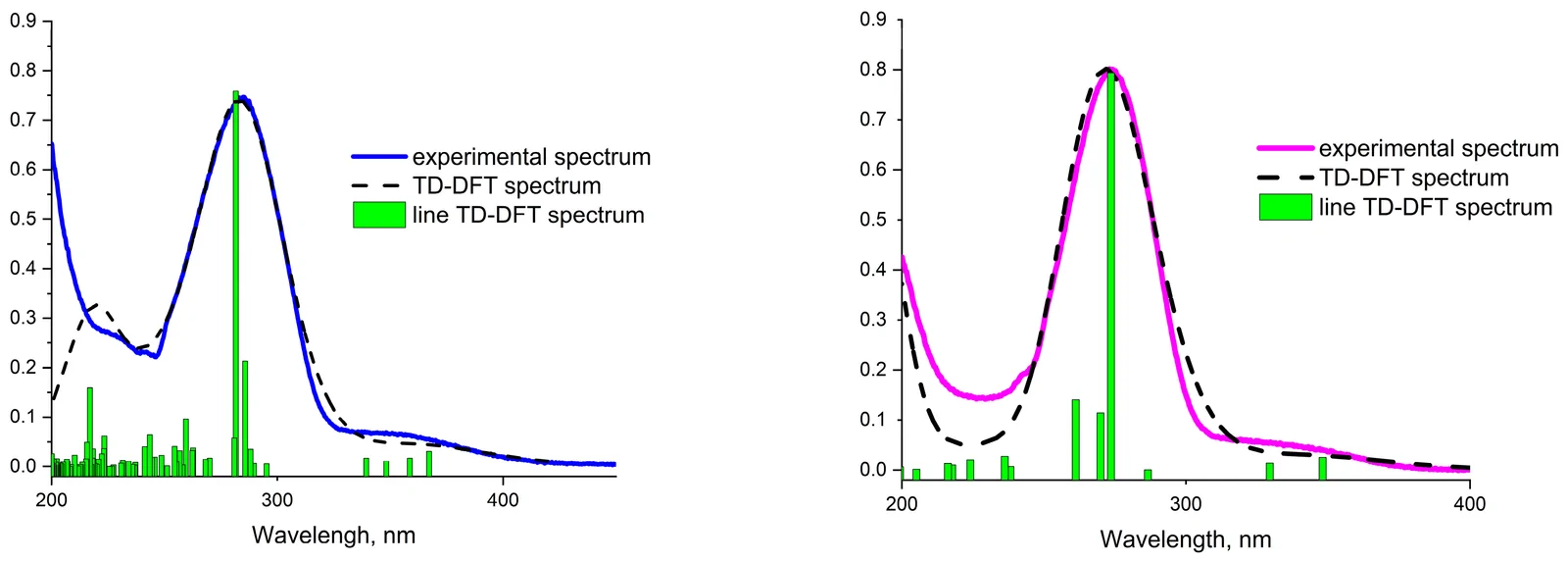
Experimental and calculated (TD-DFT) electronic absorption spectra of **3** (**left**) and L^Morph^ (**right**) in acetonitrile.

To rationalise the experimental absorption bands, TD-DFT calculations were performed for the cation of complex **3** and the ligand. Only transitions with oscillator strengths (*f*) greater than 0.05 are listed in Table 2 and further discussed. The calculated spectra are compared with the experimental ones in Figure 4.

For complex **3**, the strongest absorption band (*f* = 0.759, 4.40 eV, 282 nm) is dominated by intraligand charge transfer (ILCT) within a pyrazolyl–pyridazine moiety, with a minor contribution from interligand charge transfer (LL’CT) between two such ligands. The next band (*f* = 0.213, 4.34 eV, 286 nm) arises mainly from LL’CT between different pyrazolyl–pyridazine units. A weaker band at 4.78 eV (260 nm, *f* = 0.096) involves charge transfer from one pyrazolyl–pyridazine unit to the InCl_2_ fragment. The highest-energy band (5.71 eV, 217 nm, *f* = 0.159) has a mixed ILCT/LL’CT π–π* character and also involves a contribution from chloride ligands. In the case of the free ligand, the most intense peak (*f* = 0.635, 4.53 eV, 274 nm) corresponds to the HOMO → LUMO+1 excitation. The second transition (*f* = 0.0914, 4.59 eV, 270 nm) is mainly HOMO-3 → LUMO, while the band at 4.75 eV (261 nm, *f* = 0.113) comprises several contributions, notably, HOMO-2 → LUMO+1 and HOMO-1 → LUMO+1 (Table 2).

Overall, the TD-DFT results confirm that the observed absorption bands in both the complex and the ligand are dominated by π–π* excitations with a charge transfer character, either intra- or interligand, which is consistent with the nature of the pyrazolyl–pyridazine fragments. It is also worth noting that the HOMO → LUMO transitions, which have the lowest energies, have very weak oscillator strengths. In the case of complex **3**, this transition has a mixed ILCT/LL’CT π–π* character (*f* = 0.031, 3.13 eV, 397 nm), while, for the ligand, it occurs with an energy of 3.56 eV (*f* = 0.020, 348 nm).

In conclusion, the TD-DFT results for complexes **2** and **3** corroborate a common electronic framework based on π–π* charge transfer excitations within the pyrazolyl–pyridazine ligands. However, the observed red shift of the main band, the differing dominance of ILCT vs. LLCT, the emergence of metal-involving transitions, and the explicit participation of chloride ligands in **3** collectively indicate that the electronic structure is sensitive to subtle variations in ligand substitution, metal identity, or the coordination environment. These distinctions underscore the importance of detailed transition assignment for rationalising the photophysical behaviour of related coordination compounds.

To gain deeper insight into the nature of these transitions, particularly the role of the donor substituent in complex **3**, an electron–hole pair distribution analysis was performed for the lowest-lying S_1_ transitions in complexes **2** and **3**. It should be noted that the UV-Vis spectra for complexes **2** and **3** were calculated using different functionals, so a direct quantitative comparison of the transition energies was not carried out. Nevertheless, the electron–hole pair distribution analysis provides a reliable qualitative comparison of the transition character based on the spatial distribution of the hole and electron, as the key conclusions regarding the nature of the transitions are robust to the choice of functional [54]. The key parameters are D—the distance between the centroids of the hole and electron (the greater the distance, the stronger the charge transfer); Sr—the overlap integral of their distributions (the smaller the overlap, the stronger the separation); and t—the degree of separation (a positive value indicates charge transfer). The results of the analysis for the lowest-lying S_1_ transition are presented in Table 3.

**Figure 5:**
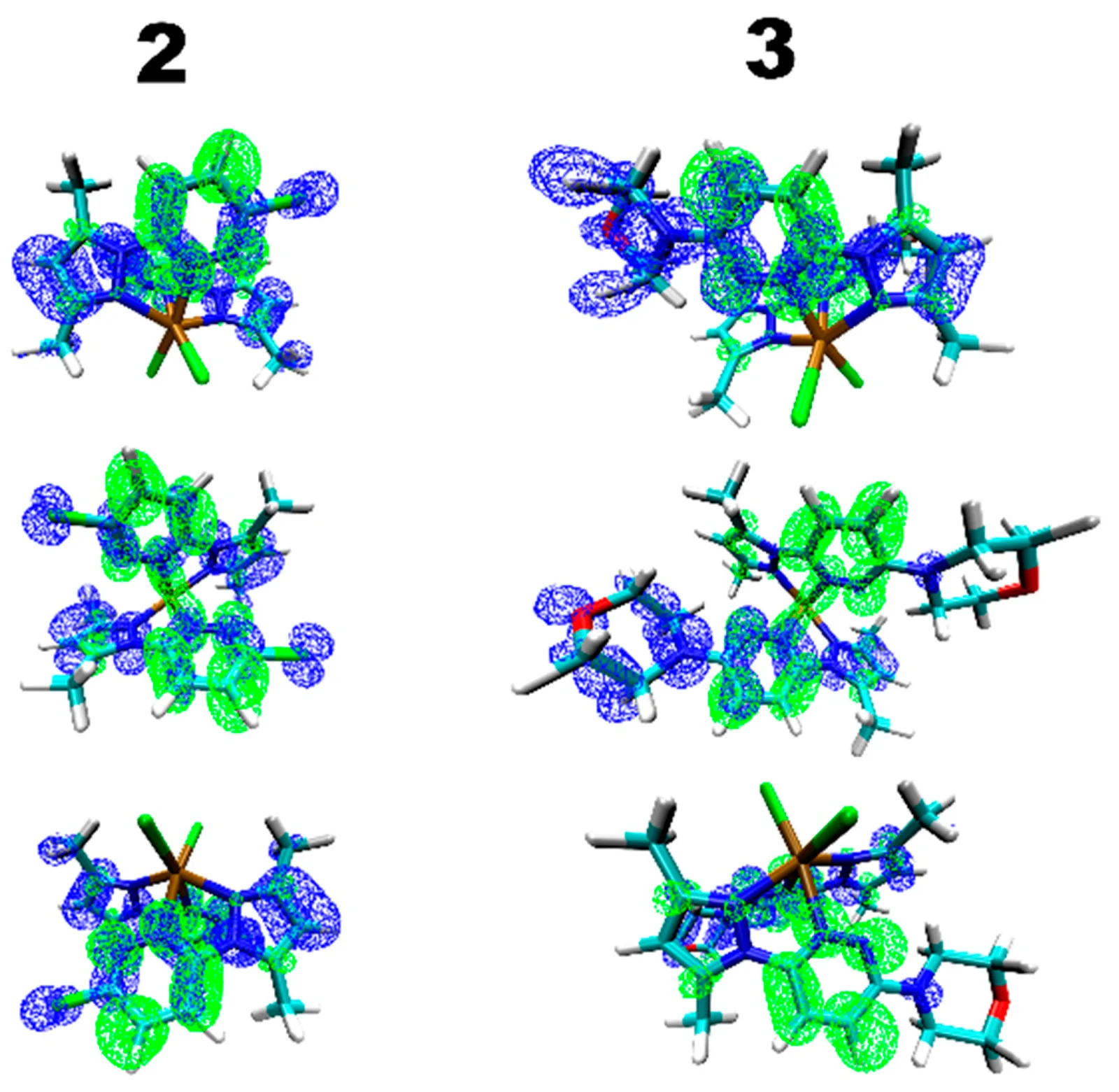
Isosurfaces of holes (blue) and electrons (green) for complexes **2** (**left**) and **3** (**right**) obtained from electron–hole analysis.

For complex **2**, which contains an electron-withdrawing chloride substituent, the distance between the centroids of the hole and electron is only 0.938 Å, and the separation index t is negative (−0.610), indicating local excitation (LE). The hole and electron occupy the same region of space and overlap strongly (Sr = 0.545). Visualisation of the isosurfaces (Figure 5, left) confirms that both isosurfaces are uniformly distributed over the two ligands, consistent with local excitation involving intraligand charge transfer (ILCT) within each ligand.

In complex **3**, which contains an electron-donating morpholine substituent, a fundamentally different picture is observed. According to the isosurfaces (Figure 5, right), the hole is delocalised over one L^Morph^ ligand, while the electron is distributed between two pyridazine moieties of different L^Morph^ ligands. The distance between the centroids increases to 2.847 Å, the separation index becomes positive (+0.759), and the overlap decreases to Sr = 0.479, indicating interligand charge transfer (LL’CT) with an accompanying intraligand charge transfer contribution (ILCT). This change in the nature of the transition is attributed to the electron-donating effect of the morpholine substituent, which increases the electron density on the pyridazine ring, promoting localisation of the HOMO on one ligand while the LUMO remains distributed over the pyridazine moieties of both ligands (Figure S6). Additional confirmation of the CT character is provided by the change in the dipole moment upon excitation: in complex **2**, this value is 4.30 D, whereas, in complex **3**, it increases to 13.39 D (Table 3). An increase by more than a factor of three indicates significantly stronger charge redistribution in complex **3**, fully consistent with its LL’CT character.

Analysis of electron–hole pairs showed that the introduction of a donor morpholine substituent instead of chloride changes the nature of the S_1_ transition from local excitation (LE) in complex **2** to interligand charge transfer (LL’CT) in complex **3**. This is confirmed by an increase in the distance between the hole and electron centroids from 0.938 to 2.847 Å and an increase in the change in dipole moment from 4.30 to 13.39 D.

**Figure 6:**
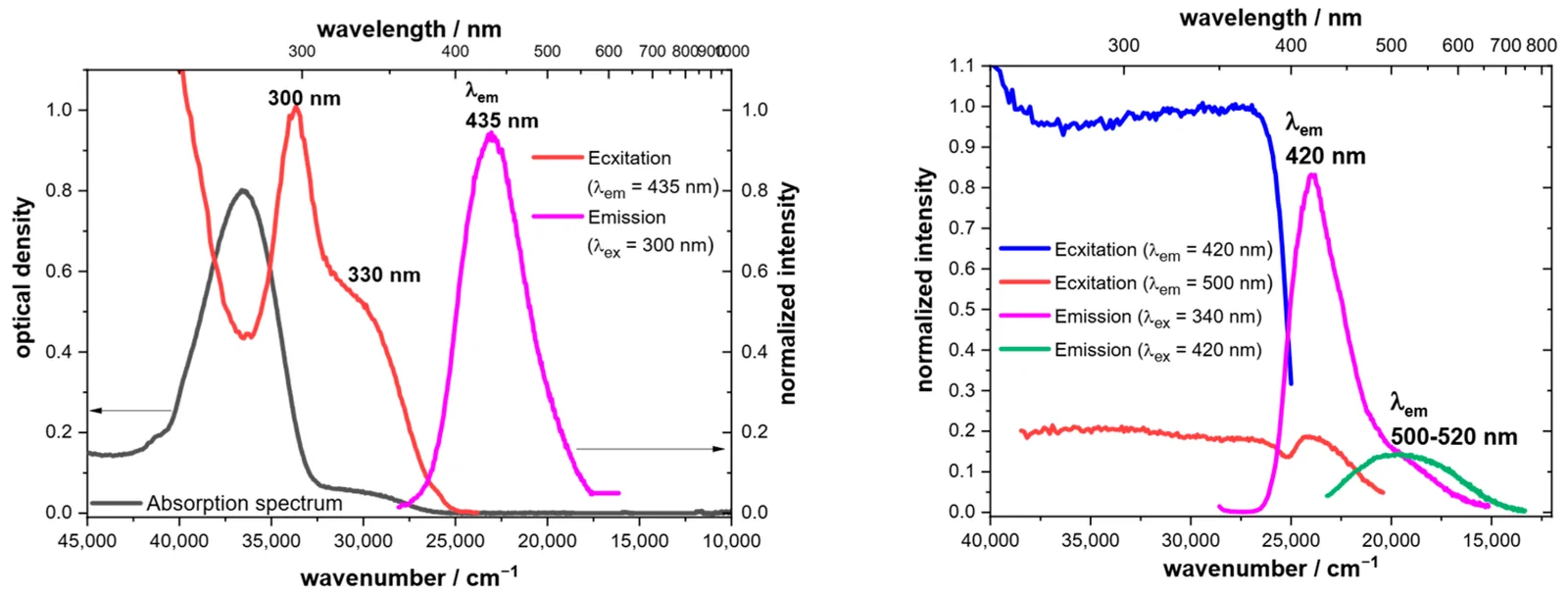
Excitation, emission, and absorption spectra of L^Morph^ in MeCN (**left**) and in the solid state (**right**).

**Figure 7:**
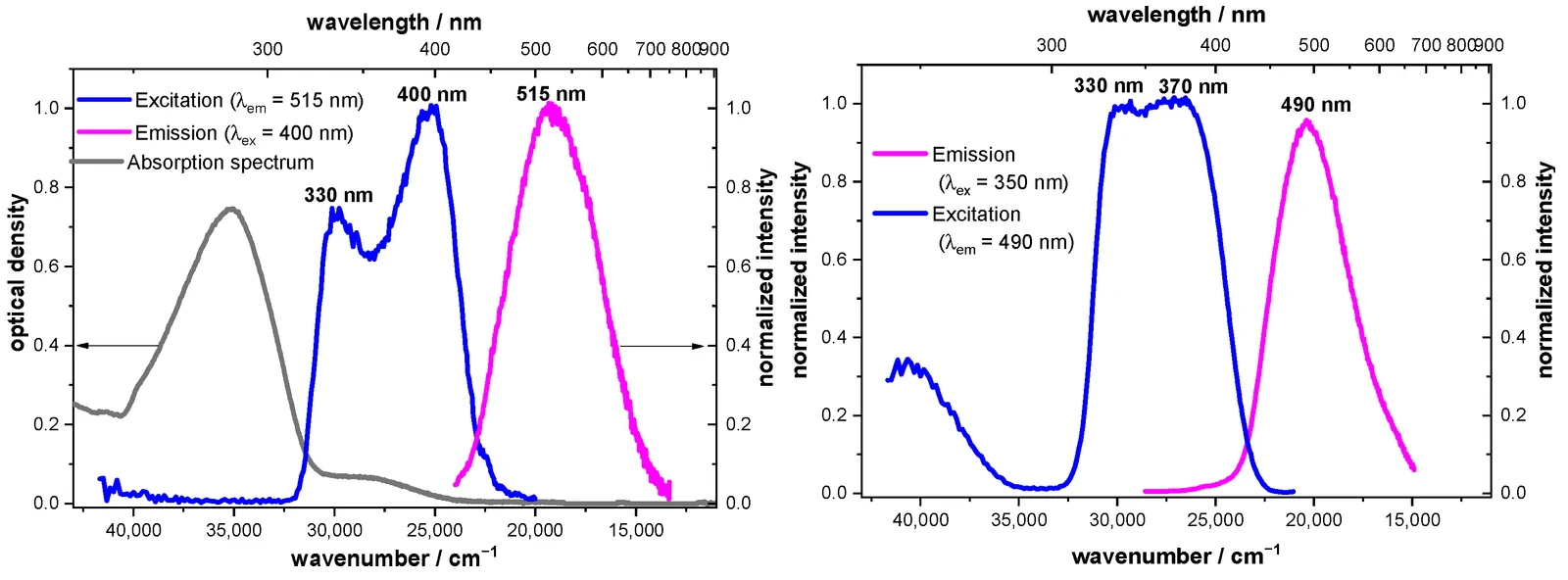
Excitation, emission, and absorption spectra of **3** in MeCN (**left**) and in CH_2_Cl_2_ (**right**).

**Figure 8:**
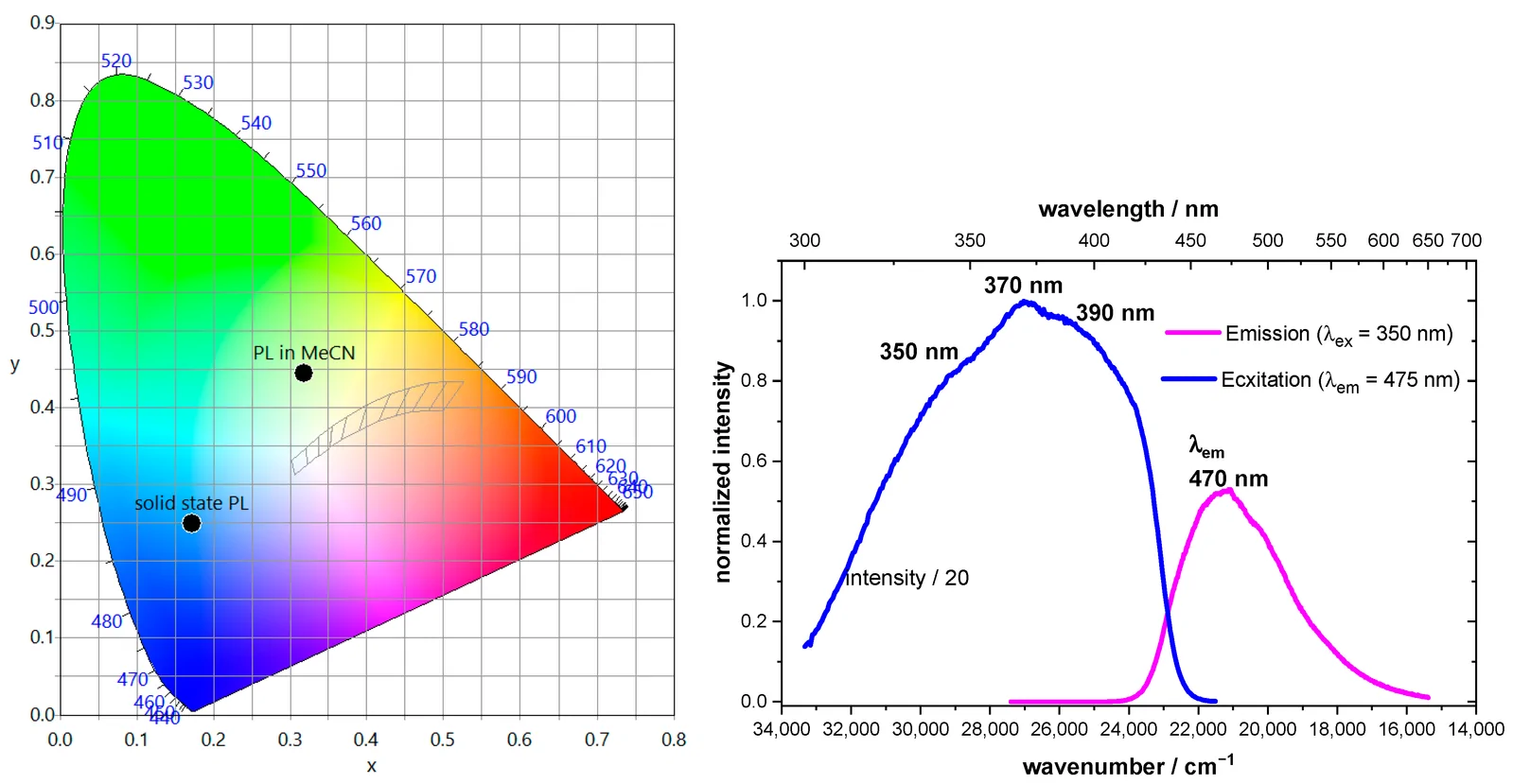
*CIE* 1931 diagram for **3** in the solid state and in solution (**left**). Excitation and emission spectra of **3** in the solid state (**right**).

The excitation and PL spectra for [In(L^Morph^)_2_Cl_2_][InCl_4_] (**3**) and L^Morph^ are presented in Figure 6, Figure 7 and Figure 8 and S7 and S8, while lifetimes and quantum yields are collected in Table 4. In the spectra of L^Morph^ in MeCN solution, the excitation maxima at 300 and 330 nm and the emission maximum at 435 nm (Figure 6 (left)) are bathochromically shifted relative to the spectrum of L^Me^ (the same trend as in the absorption spectra (see above)). However, when comparing the photophysical properties of L^Morph^ and L^Me^, no significant differences in lifetimes or quantum yields were found. The excited-state lifetimes for both compounds are around several nanoseconds, and quantum yields cannot be measured due to the weak luminescence intensity. When comparing the PL properties of [In(L^Me^)_2_Cl_2_][InCl_4_] (**2**) and **3** in MeCN solution, no significant differences in lifetimes and quantum yields were found. However, the emission band of **3** (Figure 7 (left), *CIE 1931* 0.3181, 0.4443) is significantly red-shifted by ca. 140 nm relative to the emission band of **2** (Table 4). It should be noted that **3** is almost not excited up to 300 nm, while two intense excitation bands are located in the region of 320–420 nm, where the complex has extremely low absorption. Apparently, it causes a low quantum yield of the complex in solution in contrast to the solid state (see below).

The solid-state photoluminescent properties of L^Morph^ (Figure 6 (right), Table 4) are generally similar to those of the previously published L^Me^, but that the substitution of the Cl by the electron-donating morpholino group leads to a bathochromic shift of the fluorescence bands by ca. 40–60 (420 and 500–520 nm). Furthermore, the quantum yield increases to 1.5%.

When comparing the PL properties of **3** and the L^Morph^, it was found that the PL spectrum of **3** lacks excitation-dependent emission, and the emission band is broadened and red-shifted (Figure 8 (left) and Figure S9). The CHEF effect is also clearly evident: QY increases 10 times upon complex formation. This is also confirmed by the non-radiative relaxation constants; the constant is an order of magnitude lower for the complex than for the ligand L^Morph^ (Table 4).

The same tendency in the red-shifted PL maxima is also observed when comparing the solid-state spectra of complexes **2** and **3** (Table 4). The emission of **3** is significantly red-shifted (ca. 30 nm) compared to that of **2** (Figure 8, *CIE 1931* 0.1725, 0.2487). Moreover, differences between PL properties of **2** and **3** are observed both in the excited-state lifetimes and in the quantum yields. The major lifetime of **3** is an order of magnitude longer than that for **2**. The quantum yield of **3** increases to 15%, while the quantum yield of **2** is so low that it cannot be measured.

Two interesting features of the emission maxima were found in the PL spectra of complex **3** in solution and in the solid state: (i) the emission of **3** is significantly quenched in MeCN, and (ii) the PL of **3** shows an unusual bathochromic shift of the PL band in MeCN (λ_max_ = 515 nm) in comparison with the solid-state luminescence (λ_max_ = 470 nm). This behaviour was shown to be unrelated to concentration or aggregation effects (Figure S10) and is likely due to the influence of the solvent, and possibly the presence of water in acetonitrile. Indeed, the ^1^H NMR spectrum of **3** in CD_3_CN contains an intense signal at 2.10 ppm due to the presence of water. To test this assumption, the PL spectrum of **3** was measured in dried dichloromethane (Figure 7 (right)). Replacing acetonitrile with dichloromethane resulted in a 25 nm (490 nm) hypsochromic shift of the emission band, which is closer to the PL band of the complex in the solid state. This indicates the possible involvement of water in the coordination to indium(III) ion in the solution of **3**.

## 3. Experimental Section

### 3.1. Materials and Methods

All commercially available reagents, i.e., InCl_3_·1.2H_2_O (Sigma-Aldrich, St. Louis, MO, USA, 98%), 3,6-dichloropyridazine (98%, abcr), hydrazine hydrate (98%), acetylacetone (98%), 1,1,3,3-tetramethoxypropane (99%, abcr), acetonitrile-d3 (purity 99.9%), dimethyl sulfoxide-d6 (purity 99.9%), and chloroform-d (purity 99.9%), were used as purchased. Organic solvents (acetone ((CH_3_)_2_CO), ethanol (C_2_H_5_OH), and hexane (C_6_H_14_)) were dried by standard methods. Acetonitrile (MeCN) and dichloromethane (CH_2_Cl_2_) solvents for electronic and fluorescence spectroscopy were of the HPLC purity grade and were commercially available. CH_2_Cl_2_ was further dried under molecular sieves overnight before use.

### 3.2. Physical Measurements

Elemental C, H, and N analysis was performed using a Vario MICRO cube instrument (Elementar Analysensysteme GmbH, Langenselbold, Germany) following a standard procedure. IR spectra were recorded in the 4000–400 cm^−1^ range using a PerkinElmer System 2000 FTIR spectrometer (PerkinElmer Corporation, Waltham, MA, USA) (KBr pellets). Electron absorption spectra of the solutions **3** and ligand L^Morph^ in acetonitrile were recorded using an SF-2000 spectrophotometer (OKB SPECTR, Saint Petersburg, Russian Federation). ^1^H (500 MHz) and ^13^C (125 MHz) NMR spectra were acquired using a Brucker AV-300 spectrometer and Brucker DRX-500 spectrometer (Bruker BioSpin, Billerica, MA, USA), respectively, at room temperature in CD_3_CN. The chemical shifts are given in parts per million (ppm), and the solvent spectral signal was used as the standard.

Photoluminescence (PL) and excitation spectra of **3** and L^Morph^ at room temperature in the solid state were recorded on a Fluorolog QM-75-22-C (HORIBA Scientific, Kyoto, Japan) spectrofluorometer. LED (405 nm, pulse duration 1.3 ns) was used to measure the lifetime kinetic curve for complex **3**. The quantum yield (YQ) of **3** in the solid state was measured using a QuantaPhi integration sphere (Horiba).

Photoluminescence and excitation spectra of L^Morph^ in the solid state at room temperature and complex **3** and L^Morph^ in MeCN solutions were recorded using a Horiba Fluorolog 3 spectrofluorometer equipped with steady (450 W) and pulsed xenon lamps as light sources, a cooled detector, and double grating excitation and emission monochromators. The solutions for the measurements were not degassed, and all measurements were carried out in the ambient air. The solution concentrations were as follows: 1.1 × 10^−4^ M of L^Morph^ in MeCN, 3.4 × 10^−4^ M of **3** in MeCN, 2.7 × 10^−4^ M of **3** in CH_2_Cl_2_. The photoluminescence and excitation spectra were corrected for the source intensity (lamp and grating) and to the spectral response to emission (detector and grating) using standard correction curves. The photoluminescence decay kinetics for the **3** and L^Morph^ in MeCN and for the L^Morph^ in the solid state at room temperature were recorded by time-correlated single photon counting using a NanoLED pulsed light source (HORIBA Scientific, Edison, NJ, USA) and a NanoLED-C2 controller (HORIBA Scientific, Edison, NJ, USA). Excitation and emission (registration) wavelengths for decay kinetic measurements for each compound are presented in Table 4. The quantum yield of L^Morph^ in the solid state was measured using a Fluorolog 3 spectrofluorometer equipped with a quantum sphere (Quanta-j) (HORIBA Scientific, Edison, NJ, USA), and maxima in excitation spectra were used as excitation wavelengths for YQ measurements.

### 3.3. X-Ray Diffraction Analysis

Single-crystal X-ray diffraction (XRD) data for **3** were collected using a Bruker D8 Venture diffractometer (Bruker AXS, Karlsruhe, Germany) with a CMOS PHOTON III detector (Bruker AXS, Karlsruhe, Germany) and an IμS 3.0 microfocus source (collimating Montel mirrors) (Incoatec GmbH, Geesthacht, Germany). All of the experiments were carried out at 150 K using Cu Kα (λ = 1.54184 Å) radiation. Absorption correction was applied by SADABS [55]. The structure solution was obtained by the dual-space method with SHELXT-2018/2, followed by refinement against |F|^2^ using SHELXL-2019/2 [56] and ShelXle GUI (version 1.0.1556) [57]. Atomic displacement parameters for non-hydrogen atoms were refined anisotropically. Hydrogen atoms were placed in geometrically calculated positions and refined using the riding model.

Crystallographic data and details of the diffraction experiments for complex **3** are presented in Table S1. The crystallographic data were deposited in the Cambridge Crystallographic Data Centre under the deposition codes CCDC 2578695 (**3**). These data can be obtained free of charge from the Cambridge Crystallographic Data Centre at https://www.ccdc.cam.ac.uk/structures/ accessed on 4 August 2026.

### 3.4. Quantum Chemical Calculations

Quantum chemical calculations were performed using the ORCA 6.0.0 software package [58,59]. The crystal structure data for **3** and ligand were used as the initial points for the geometry optimisation process. Calculations were conducted within the framework of density functional theory (DFT), employing the hybrid B3LYP functional [60] with the D4 empirical dispersion correction [61]. All atoms were described with the def2-TZVP basis set. Solvent effects were modelled via the CPCM approach. TD-DFT excitation energies were computed using the ORCA implementation [62]. For the complex, all calculated transition energies were uniformly shifted by +0.25 eV to improve agreement with the experimental maxima. The electron–hole pair distribution analysis was performed using the Multiwfn 2026.8.19 program [63].

### 3.5. Synthesis of 4-(6-(3,5-Dimethyl-1H-pyrazol-1-yl)pyridazin-3-yl)morpholine (L^Morph^)

3-Chloro-6-(3,5-dimethyl-1*H*-pyrazol-1-yl)pyridazine was prepared as reported in [36]. In a 10 mL vial, 3-chloro-6-(3,5-dimethyl-1*H*-pyrazol-1-yl)pyridazine (500.0 mg, 2.400 mmol) was suspended in 3 mL of morpholine. The mixture was heated at 110 °C with stirring. After 30 min, the reaction mixture solidified, heating was continued for 1 h, and then it was cooled to room temperature. Water (7 mL) was added and the resulting mixture was transferred on a glass filter and additionally washed with water until the filtrate reached a neutral pH and dried. The yield of yellowish solid was 484.7 mg (78%). The following values were found for C_13_H_17_N_5_O: C 60.2, H 6.8, N 26.7. The following values were calculated for C_13_H_17_N_5_O: C 60.2, H 6.6, N 27.0. The melting point was 123–125 °C.

IR (KBr, cm^−1^): 3143 (w), 3100 (w), 3076 (w), 2983 (w), 2955 (m), 2935 (w), 2898 (w), 2873 (w), 2853 (s), 1605 (m), 1572 (s), 1545 (s), 1472 (s), 1447 (s), 1405 (m), 1373 (m), 1358 (m), 1337 (m), 1322 (m), 1304 (w), 1276 (m), 1263 (s), 1252 (s), 1223 (w), 1200 (w), 1179 (w), 1167 (w), 1155 (w), 1115 (s), 1072 (m), 1057 (m), 1025 (m), 1009 (m), 984 (m), 929 (s), 853 (w), 832 (s), 814 (m), 751, 667 (m), 628 (w), 593 (w), 563 (w), 529 (w), 458 (w), 436 (w), 415 (w).

^1^H NMR (CD_3_CN, δ, ppm) 7.75 (d, ^3^*J*_HH_ = 9.7 Hz, 1H), 7.21 (d, ^3^*J*_HH_ = 9.7 Hz, 1H), 6.04 (s, 1H), 3.78 (m, 4H), 3.56 (m, 4H), 2.53 (s, 3H), 2.20 (s, 3H). ^13^C1 NMR (CD_3_CN, δ, ppm) 160.2, 151.7, 150.5, 141.9, 123.6, 116.7, 109.3, 67.1, 46.3, 14.1, 13.6.

### 3.6. Synthesis of Complex [In(L^Morph^)_2_Cl_2_][InCl_4_] (**3**)

InCl_3_·1.2H_2_O (93.1 mg, 0.38 mmol) was dissolved in 15 mL ethanol. The ligand (100 mg, 0.38 mmol) was dissolved in chloroform, and then the two solutions were mixed. The resulting clear liquid was refluxed for 10 h. After cooling, the colourless liquid was evaporated to dryness, washed several times with diethyl ether, and air-dried. A white precipitate was obtained (150 mg, 81%). Colourless crystals suitable for X-ray analysis were obtained by layering hexane on a solution of the complex in chloroform. The following values were found for C_26_H_34_Cl_6_In_2_N_10_O_2_: C 32.6, H 3.6, N 14.6. The following values were calculated for C_26_H_34_Cl_6_In_2_N_10_O_2_: C 32.5, H 3.6, N 14.6.

IR (KBr, cm^−1^): 3118 (w), 2965 (w), 2902 (w), 2856 (w), 1610 (m), 1574 (m), 1544 (m), 1494 (vs), 1481 (vs), 1445 (vs), 1424 (s), 1361 (m), 1308 (w), 1292 (w), 1262 (vs), 1188 (w), 1165 (w), 1145 (w), 1116 (s), 1076 (m), 996 (m), 939 (m), 860 (w), 843 (w), 823 (s), 790 (m), 757 (w), 674 (w), 651 (w), 627 (w), 564 (w), 538 (w), 468 (w), 428 (w).

^1^H NMR (CD_3_CN, δ, ppm): 7.92 (d, ^3^J_HH_ 10.1 Hz, 1H), 7.38 (d, ^3^J_HH_ 10.1 Hz 1H), 6.49 (br.s, 1H), 3.65 (m, 4H), 3.32 (m, 4H), 2.66 (s, 3H), 2.63 (d, 3H).

## 4. Conclusions

In summary, this study demonstrates that (1*H*-pyrazol-1-yl)pyridazine ligands serve as a versatile platform for the synthesis of indium(III) complexes under mild aerobic conditions. The nature and position of substituents on the heterocyclic backbone critically determine the complex stoichiometry: the L^H^ ligand affords the neutral octahedral complex [In(L^H^)(H_2_O)Cl_3_] (**1**), while methyl and morpholine substituents favour the formation of ionic bis-chelate species [In(L)_2_Cl_2_][InCl_4_] (**2** and **3**). Structural analysis revealed that both ionic complexes adopt a *cis*-[In(L)_2_Cl_2_]^+^ cation with a tetrahedral [InCl_4_]^−^ counterion, with In–N (2.255–2.314 Å) and In–Cl (2.38–2.40 Å) distances remaining largely unaffected by peripheral substitution. However, conformational flexibility of the chelate ligands is evident from variations in the pyrazole–pyridazine dihedral angles, which appear to be governed by crystal packing rather than electronic effects. Distinct supramolecular arrangements are observed: neutral complex **1** is stabilised by O–H···Cl hydrogen bonds, while ionic complexes **2** and **3** rely on weaker interactions, with **3** additionally featuring H–π stacking (3.41 Å) mediated by lattice chloroform molecules.

The photophysical properties of the new ligand L^Morph^ and its indium(III) complex [In(L^Morph^)_2_Cl_2_][InCl_4_] (**3**) were investigated both in solution and in a solid state and compared with those of the previously reported L^Me^ and [In(L^Me^)_2_Cl_2_][InCl_4_] (**2**). The electron-donating morpholine substituent was found not to alter the nature of the luminescence, with both compounds exhibiting fluorescence with nanosecond lifetimes. However, a pronounced donor substituent effect was observed on the absorption, excitation, and emission band positions for both the free ligand and the complex. Both in solution and in the solid state, all bands undergo a bathochromic shift, with the emission band of **3** in solution being red-shifted by up to 140 nm relative to the L^Me^-based analogue **2**. A marked difference was also noted in the solid-state emission quantum yields: the quantum yield of L^Morph^ (1.5%) slightly increases compared to L^Me^ (<1%), while that of **3** substantially increases, reaching 15%.

The electron–hole analysis revealed that replacing the electron-withdrawing chloride substituent in complex **2** with the electron-donating morpholine substituent in complex **3** fundamentally changes the character of the S_1_ transition from local excitation (LE) to interligand charge transfer (LL’CT) with an intraligand contribution (ILCT). This change is accompanied by a marked increase in the dipole moment change upon excitation from 4.30 D in **2** to 13.39 D in **3**, indicating significantly stronger charge redistribution. These theoretical findings provide a direct explanation for the experimentally observed bathochromic shift and the substantial enhancement of the quantum yield.

Overall, the introduction of a donor substituent addresses a key challenge by significantly enhancing the fluorescence intensity of the indium(III) complex. A possible strategy for obtaining phosphorescent emitters based on indium(III) complexes could involve a transition to push–pull systems (D–A or D–A–D architectures), for instance, by appending an electron-withdrawing group to the ligand. Such an approach may lead to complexes exhibiting thermally activated delayed fluorescence (TADF), which would ultimately enable intense photoluminescence with prolonged lifetimes. Overall, slight modifications to the ligand periphery enable selective access to either neutral mononuclear or ionic bis-chelate architectures, offering a rational strategy for designing indium(III) coordination compounds with tunable structural motifs and photophysical properties.

## Figures and Tables

**Table 1:** Selected bond lengths in complex **3**.

Bond Type	Distance, Å
In–Cl1	2.413(1)
In–Cl2	2.390(1)
In–N1	2.278(3)
In–N2	2.294(3)
In–N1’	2.255(3)
In–N2’	2.310(3)

**Table 2:** Summary of the main energy, wavelength, and oscillator strength computed for observed transitions in the absorption spectra of **3** and L^Morph^, together with the orbitals implied.

Complex	Energy, eV	λ, nm	*f*	Major Contributions	Character
**3**	4.34	286	0.213	HOMO-1 → LUMO+2 (15%) HOMO → LUMO+3 (74%)	π(L) → π*(L) π(L) → π*(L’)
	4.40	282	0.759	HOMO-1 → LUMO+2 (76%) HOMO → LUMO+3 (14%)	π(L) → π*(L) π(L’) → π*(L’)
	4.78	260	0.096	HOMO → LUMO+4 (90%)	π(L’) → π*+σ*(In + Cl1 + Cl2)
	5.71	217	0.159	HOMO-5 → LUMO+2 (9%) HOMO-1 → LUMO+5 (6%) HOMO → LUMO+5 (8%) HOMO → LUMO+6 (56%)	π(L + L’ + Cl1 + Cl2) → π*(L) π(L) → π*(L) π(L’) → π*(L’) π(L) → π*(L)
**L^Morph^**	4.53	274	0.6353	HOMO → LUMO+1 (86%)	π → π*
	4.59	270	0.0914	HOMO-3 → LUMO (86%) HOMO-2 → LUMO (10%)	π → π*
	4.75	261	0.1125	HOMO-3 → LUMO (8%) HOMO-2 → LUMO+1 (22%) HOMO-1 → LUMO+1 (57%) HOMO → LUMO+1 (10%)	π → π*

**Table 3:** Summary of electron–hole analysis indices for complexes **2** and **3**.

Parameter	2	3
**Energy, eV**	4.01	3.13
***f***	0.103	0.031
**D, Å**	0.938	2.847
**S** **r**	0.545	0.479
**t, Å**	−0.610	+0.759
**Δμ, D**	4.30	13.39

**Table 4:** Photophysical data for L^Morph^, L^Me^ [37], **2** [36], and **3** in MeCN solution and in the solid state at room temperature.

	*In MeCN Solution* (300 *K*)					*Solid State* (300 *K*)				
	Absorption Spectra *λ* (nm)	Excitation Spectra *λ* (nm)	Emission Spectra *λ* (nm)	Lifetimes, τ (ns)	Excitation Spectra *λ* (nm)	Emission Spectra *λ* (nm)	Lifetimes, τ (ns)	Quantum Yield, φ (%)	K_r_	K_nr_
**L^Morph^**	274, 317–370	300, 330	435	1.5 ns λ_ex_ = 300 nm, λ_em_ = 435 nm	~300–370	420	2.2 ns λ_ex_ = 350 nm, λ_em_ = 420 nm	1.5	6.8 × 10^6^	4.5 × 10^8^
					420	500–520	1.1 ns (62%), 6.1 ns (38%) λ_ex_ = 350 nm, λ_em_ = 520 nm			
**3**	286, 348–400	330, 400	430	2.3 ns λ_ex_ = 350 nm, λ_em_ = 430 nm	350, 370, 390	470	23 ns λ_ex_ = 370 nm, λ_em_ = 470 nm	15	6.5 × 10^6^	3.7 × 10^7^
			515	14 ns (13%), 1.4 ns (87%) λ_ex_ = 350 nm, λ_em_ = 515 nm						
**L^Me^**	260, ~300 (sh.)	270	390	2.7 ns λ_ex_ = 300 nm, λ_em_ = 390 nm	~300–320	375	6.0 ns λ_ex_ = 300 nm, λ_em_ = 375 nm	<1	-	-
					360	450	6.5 ns λ_ex_ = 350 nm, λ_em_ = 450 nm			
**2**	260, 290–300 (sh.)	260, 290–300 (sh.)	375	6.4 ns (94%), 2.6 ns (6%) λ_ex_ = 300 nm, λ_em_ = 375 nm	~300–330	375	19 ns (9%), 1.4 ns (91%) λ_ex_ = 300 nm, λ_em_ = 375 nm	<1	-	-
					350, 380	440	16 ns (10%), 3.8 ns (90%) λ_ex_ = 350 nm, λ_em_ = 440 nm	<1		
						500	-			

## Data Availability

The crystallographic data have been deposited in the Cambridge Crystallographic Data Centre under the deposition code CCDC 2578695 (**3**).

## Supplementary Materials

The following supporting information can be downloaded at https://www.mdpi.com/article/10.3390/molecules31183217/s1, Table S1. SCXRD experimental details for **3**·CHCl_3_. Table S2. NMR data for complex **3** and free L^Morph^. Figure S1. The crystal packing of **3**·CHCl_3_. Figure S2. NMR spectrum of free ligand L^Morph^ in CD_3_CN. Figure S3. NMR spectrum of complex **3** in CD_3_CN. Figure S4. X-ray Powder diffraction spectra of complex [In(L^Morph^)_2_Cl_2_][InCl_4_] (**3**): experimental (black line) and theoretical (red line). Figure S5. The electronic absorption spectra of L^Morph^ (black line) and **3** (red line) in acetonitrile. Figure S6. HOMO and LUMO isosurfaces of complexes **2** (left) and **3** (right), calculated at the PBE0(D4)/def2-TZVP and B3LYP(D4)/def2-TZVP levels, respectively. Figure S7. Excitation, emission and absorption spectra of **3** in MeCN. Figure S8. Excitation, emission and absorption spectra of L^Morph^ in MeCN including PL bands at various excitation wavelength. Figure S9. Excitation and emission spectra of **3** in the solid state. Figure S10. PL spectra of **3** at different concentration in the MeCN solution.
