# Supplementary material for: Luminescence Enhancement of an Indium(III) Complex Through Introducing an Electron-Donating Group in (1H-Pyrazol-1-yl)pyridazine
Source: Molecules. 2026 Sep 11;31(18):3217. doi: 10.3390/molecules31183217 (PMC13609678; doi:10.3390/molecules31183217)
Supplement: Supplementary file 1 [file molecules-31-03217-s001.zip › molecules-4516886-supplementary.pdf]

Supplementary Materials:

Luminescence enhancement of Indium(III) complex by introducing electron-donating group in (1*H*-pyrazol-1-yl)pyridazine

Evgeniia Sergeevna Sedykh [1], Nikita Vasilievich Naletov [1], Veronika Igorevna Komlyagina [2], Yulia Sergeevna Spiridonova [3], Elvira Ilgizovna Musina [3], Marianna Ivanovna Rakhmanova [2], Nikolay Filippovich Romashev [2], Katerina Aleksandrovna Vinogradova [2], Marat Damirovich Nafikov [1,2], Alexey Yuryevich Vorob'ev [4], Iakov Sergeevich Fomenko [2,*] and Artem Leonidovich Gushchin [2,*]

Content

Table S1. SCXRD experimental details for **3**·$CHCl_3$

Figure S1. The crystal packing of **3**·$CHCl_3$

Table S2. NMR data for complex **3** and free L^{Morph}

Figure S2 NMR spectrum of free ligand L^{Morph} in CD_3CN

Figure S3. NMR spectrum of complex **3** in CD_3CN

Figure S4. X-ray Powder diffraction spectra of complex $[In(L^{Morph})_2Cl_2][InCl_4]$ (**3**): experimental (black line) and theoretical (red line)

Figure S5. The electronic absorption spectra of L^{Morph} (black line) and **3** (red line) in acetonitrile

Figure S6. HOMO and LUMO isosurfaces of complexes **2** (left) and **3** (right), calculated at the PBE0(D4)/def2-TZVP and B3LYP(D4)/def2-TZVP levels, respectively.

Figure S7. Excitation, emission and absorption spectra of **3** in MeCN.

Figure S8. Excitation, emission and absorption spectra of L^{Morph} in MeCN including PL bands at various excitation wavelength.

Figure S9. Excitation and emission spectra of **3** in the solid state.

Figure S10. PL spectra of **3** at different concentration in the MeCN solution.

Table S1. SCXRD experimental details for **3**·$CHCl_3$

Crystal data	
Chemical formula	$C_{26}H_{34}Cl_2InN_{10}O_2Cl_4In{\cdot}CHCl_3$
M_r	1080.34
Crystal system, space group	Orthorhombic, *Pbca*
Temperature (K)	220
a, *b*, *c* (Å)	18.8991 (10), 15.3683 (8), 28.4471 (14)
V (Å^3)	8262.4 (7)
Z	8
F(000)	4272
Radiation type	Cu $K\alpha$
μ (mm^{-1})	14.62
Crystal size (mm)	0.28 × 0.12 × 0.07
Data collection	
Diffractometer	Bruker D8 Venture diffractometer
Absorption correction	Multi-scan<br>*SADABS* 2016/2: Krause, L., Herbst-Irmer, R., Sheldrick G.M. & Stalke D., J. Appl. Cryst. 48 (2015) 3-10
T_{min}, T_{max}	0.220, 0.754
No. of measured, independent and observed [$I > 2\sigma(I)$] reflections	48406, 8505, 7552
R_{int}	0.095
θ values (°)	θ_{max} = 78.5, θ_{min} = 3.1
$(\sin \theta/\lambda)_{max}$ (Å^{-1})	0.636
Range of *h*, *k*, *l*	$h = -23 \rightarrow 22$, $k = -19 \rightarrow 19$, $l = -32 \rightarrow 34$
Refinement	
$R[F^2 > 2\sigma(F^2)]$, $wR(F^2)$, S	0.048, 0.131, 1.05
No. of reflections	8505
No. of parameters	520
No. of restraints	120
H-atom treatment	H-atom parameters constrained
$\Delta\rho_{max}$, $\Delta\rho_{min}$ (e Å^{-3})	1.97, −0.99

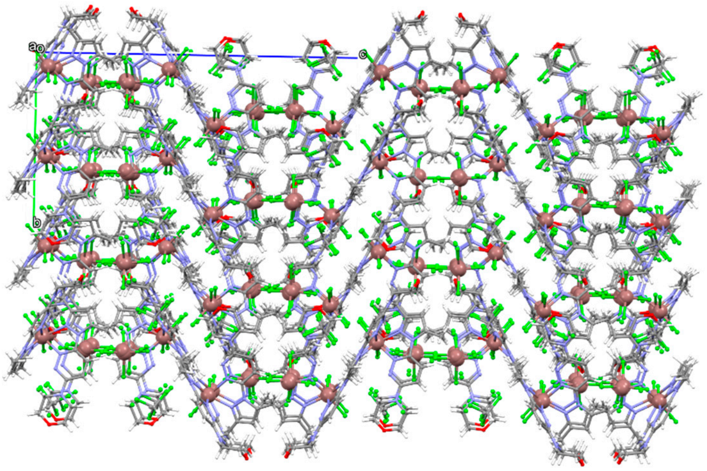

Figure S1. The crystal packing of **3**·$CHCl_3$

Table S2. NMR data for complex **3** and free L^{Morph}

Compound	Pyridazine H (ppm)	Pyrazole H4 (ppm)	Morpholine – O–CH$_2$– (ppm)	Morpholine – N–CH$_2$– (ppm)	CH$_3$ (ppm)
**L^{Morph}**	7.74, 7.21	6.03	3.77	3.55	2.52, 2.20
**3**	7.92, 7.37	6.49	3.65	3.32	2.63

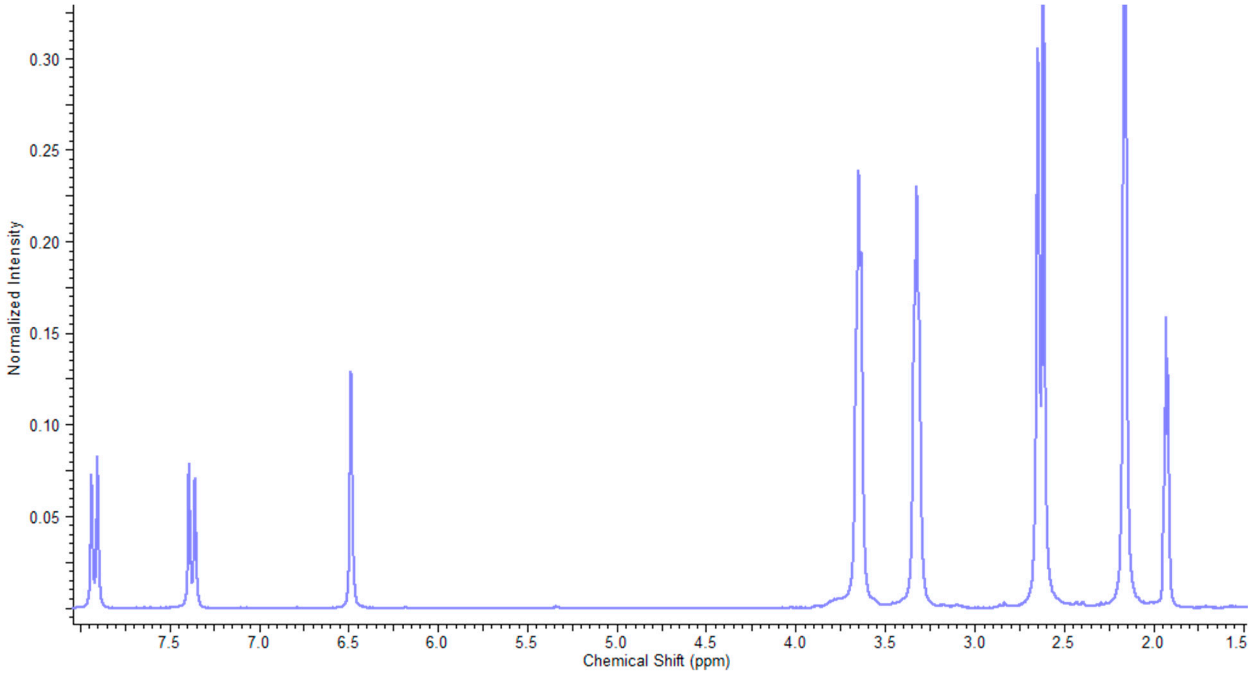

Figure S2. NMR spectrum of complex **3** in CD_3CN

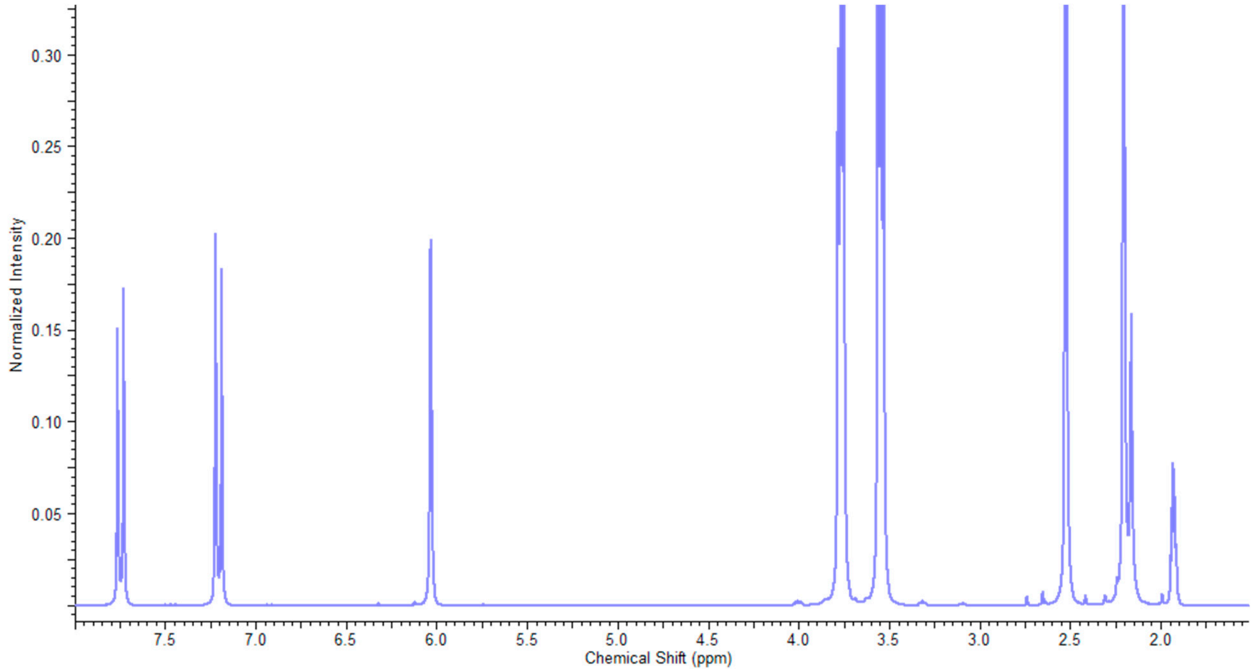

Figure S3. NMR spectrum of free ligand L^{Morph} in CD_3CN

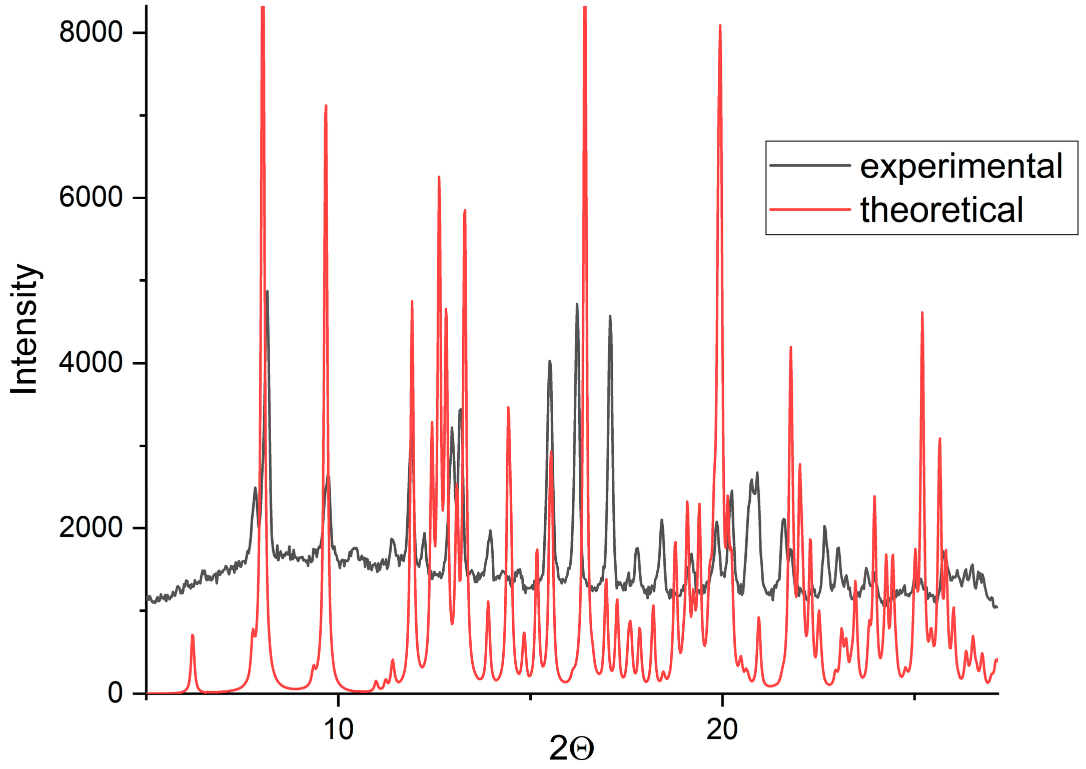

Figure S4. X-ray Powder diffraction spectra of complex $[In(L^{Morph})_2Cl_2][InCl_4]$ (**3**): experimental (black line) and theoretical (red line)

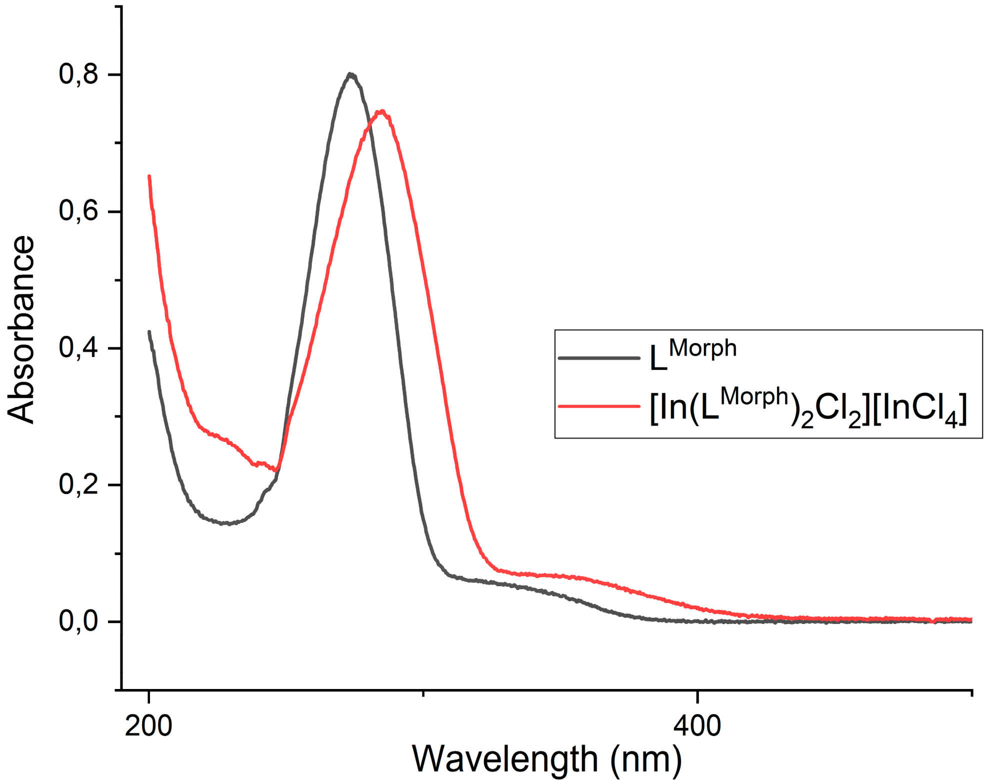

Figure S5. The electronic absorption spectra of L^{Morph} (black line) and **3** (red line) in acetonitrile

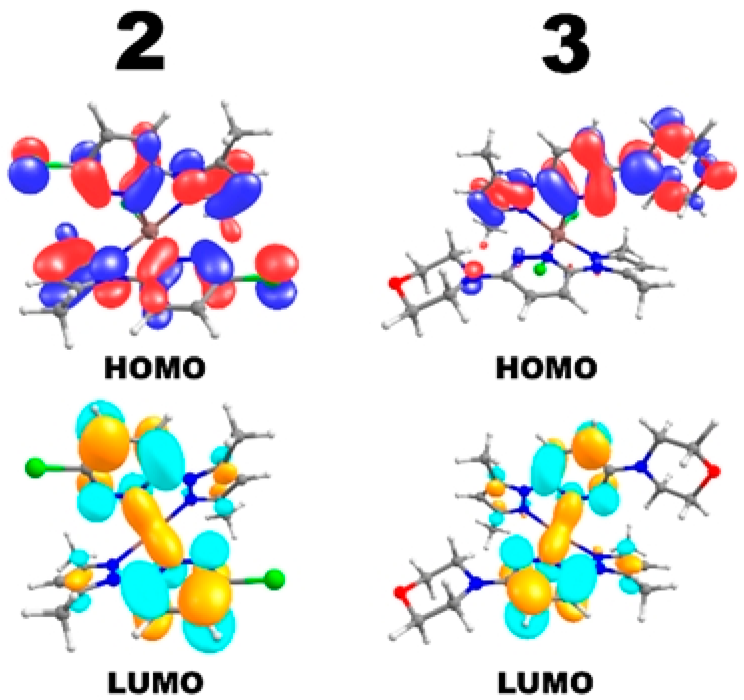

Figure S6. HOMO and LUMO isosurfaces of complexes **2** (left) and **3** (right), calculated at the PBE0(D4)/def2-TZVP and B3LYP(D4)/def2-TZVP levels, respectively.

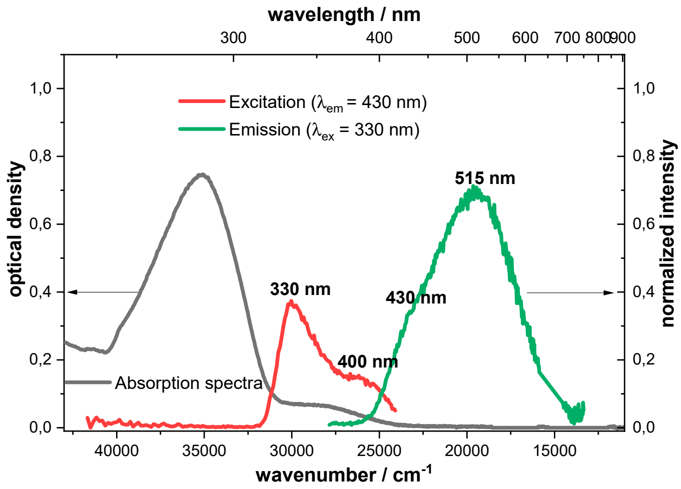

Figure S7. Excitation, emission and absorption spectra of **3** in MeCN.

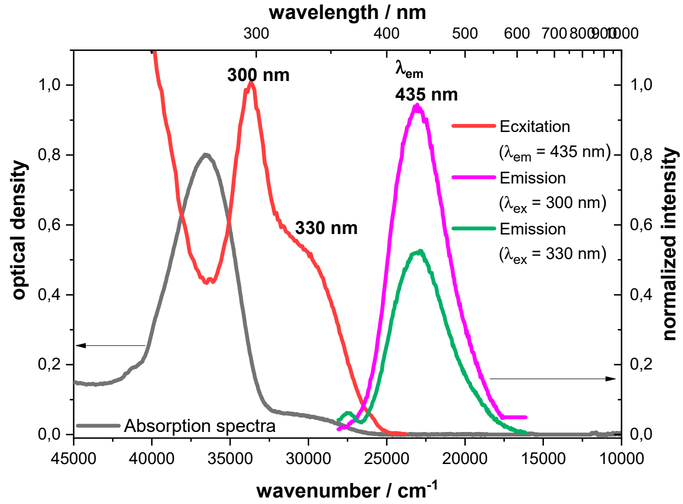

Figure S8. Excitation, emission and absorption spectra of L^{Morph} in MeCN including PL bands at various excitation wavelength.

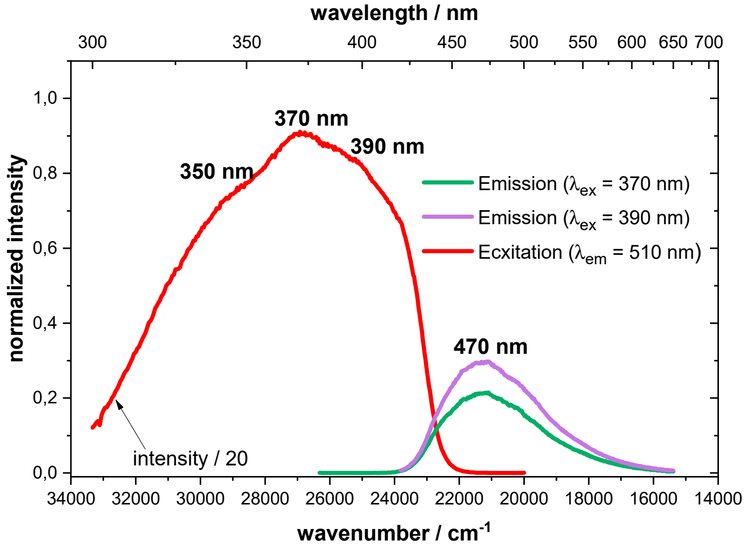

Figure S9. Excitation and emission spectra of **3** in the solid state.

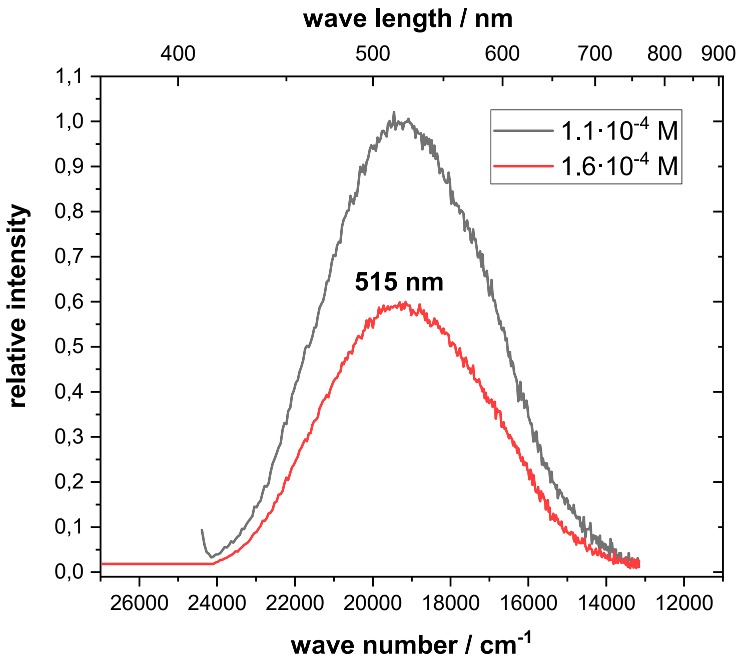

Figure S10. PL spectra of **3** at different concentration in the MeCN solution.
